# Supplementary material for: Skin-like cryogel electronics from suppressed-freezing tuned polymer amorphization
Source: Nat Commun. 2023 Aug 17;14:5010. doi: 10.1038/s41467-023-40792-y (PMC10435499; doi:10.1038/s41467-023-40792-y)
Supplement: Supplementary file 1 — Supplementary Information [file 41467_2023_40792_MOESM1_ESM.pdf]

Supplementary Information

Skin-like cryogel electronics from suppressed-freezing tuned polymer amorphization

Xiansheng Zhang[1,10], Hongwei Yan[2,10], Chongzhi Xu[3,10], Xia Dong[4], Yu Wang[4], Aiping Fu[1], Hao Li[5], Jin Yong Lee[5], Sheng Zhang[6], Jiahua Ni[7], Min Gao[8], Jing Wang[8], Jinpeng Yu[2], Shuzhi Sam Ge[9], Ming Liang Jin[2]*, Lili Wang[1]* & Yanzhi Xia[1]

[1]State Key Laboratory of Bio-Fibers and Eco-Textiles, College of Textiles and Clothing, Qingdao University, Qingdao 266071, China.
[2]Institute for Future, Shandong Key Laboratory of Industrial Control Technology, School of Automation, Qingdao University, Qingdao 266071, China.
[3]College of Materials Science and Engineering, Qingdao University, Qingdao 266071, China.
[4]CAS Key Laboratory of Engineering Plastics, Institute of Chemistry, Chinese Academy of Sciences, Beijing 100190, China.
[5]Department of Chemistry, SungKyunKwan University, Suwon 16419, Korea.
[6]State Key Laboratory of Fluid Power and Mechatronic Systems, College of Mechanical Engineering, Zhejiang University, Hangzhou 310027, China.
[7]College of Biological Science and Medical Engineering, Donghua University, Shanghai 201620, China.
[8]Institute of Environmental Engineering, ETH Zürich, Zürich 8093, Switzerland.
[9]Department of Electrical and Computer Engineering, National University of Singapore, 117576, Singapore
[10]These authors contributed equally: Xiansheng Zhang, Hongwei Yan, Chongzhi Xu.
*email: jinmingliang@qdu.edu.cn; llwang@qdu.edu.cn

List of Supplementary Notes and Figures

Supplementary Note 1. Measurements of freezing temperature for precursor solution, transmittance and water retention ability of hydrogels

Supplementary Note 2. Details of molecular dynamics simulations

Supplementary Note 3. The ionic-conductivity of suppressed cryogels

Supplementary Note 4. Electrochemical measurements of the suppressed cryogel electrodes

Supplementary Fig. 1 The freezing behaviors of precursor solution with anti-freezing salts.

Supplementary Fig. 2 The SAXS patterns of cryogels and suppressed cryogels.

Supplementary Fig. 3 Molecular dynamics simulations of PVA chains surrounding by various number of water molecules.

Supplementary Fig. 4 The transparency of cryogels with various concentrations of $CaCl_2$ in precursor solution.

Supplementary Fig. 5 The mechanical behaviors of suppressed cryogels.

Supplementary Fig. 6 The variations of tensile and self-adhesive behaviors with fabricating parameters.

Supplementary Fig. 7 The self-adhesive nature of suppressed cryogels.

Supplementary Fig. 8 The schematic illustration of self-adhesiveness and its variation with the distance between adjacent chains *d*.

Supplementary Fig. 9 The schematic mechanism and the corresponding appearance of self-healing for suppressed cryogels and conventional freeze-thawed cryogels.

Supplementary Fig. 10 Photographs showing reprocessing ability into arbitrary shapes for suppressed cryogels.

Supplementary Fig. 11 The stability of suppressed cryogels in comparison with conventional freeze-thawed cryogels.

Supplementary Fig. 12 The conductivity of suppressed cryogels.

Supplementary Fig. 13 The resistance changes of hydrogels with the same size under different cutting conditions.

Supplementary Fig. 14 Information transmission performance of artificial nerve fibers when stretched at low temperature.

Supplementary Fig. 15 Simulation of the entire module when the hand is in contact with the hydrogel.

Supplementary Fig. 16 The comparison of specific position between the self-healing touch pad and the computer.

Supplementary Fig. 17 The impedance and resistance changes of the pressure sensor under sweep and fixed frequency.

Supplementary Fig. 18 Electrochemical performance of the suppressed cryogel electrodes with different $CaCl_2$ concentrations.

Supplementary Fig. 19 Sensitivity of the pressure sensor.

Supplementary Fig. 20 Response time, stability and repeatability of pressure sensors.

Supplementary Fig. 21 Fabrication process of pressure sensor and pressure sensor array.

Supplementary Note 1. Measurements of freezing temperature for precursor solution, transmittance and water retention ability of hydrogels

The freezing temperature (T_f) of precursor solution was determined using differential scanning calorimeter (DSC) analysis. The precursor solution with weight of ~ 10 mg was added into Aluminum pan and then underwent the following thermal runs. First, it was cooled to –80 °C at 1 °C min^{-1}, then kept at –80 °C for 30 min, and finally heated to 40 °C at 1 °C min^{-1}. The cooling and heating runs were recorded.

The visible light transmittance of the hydrogel was measured by a UV-visible spectrophotometer. The thickness of the hydrogel sample is 2 mm. The wavelength range is 200–800 nm, and the transmittance within 400–800 nm is analyzed.

A constant temperature and humidity chamber was used to analyze the water retention ability of the hydrogels. The weight changes of the hydrogels (T = 15 °C and RH = 60%) were recorded for every 3 h for 160 h.

Supplementary Note 2. Details of molecular dynamics simulations

In order to investigate the conformation of polymer chains and the corresponding H-bonding interaction between PVA-PVA chains after FT process, molecular dynamics (MD) simulations were performed using Forcite module in Materials Studio. For conventional FT process, since that most of water molecules tend to arrange into ice with large size, the number of water molecules interacting with PVA chains is less. However, the SFT process allows more water molecules to interact with polymer chains due to the anti-freezing ability of $CaCl_2$. Therefore, the MD simulations were carried out under different number of water molecules. To simplify the simulation system, the initial structures are composed of three parallel fully extended PVA chains, in which the number of water molecules is varied (1.0, 0.1 and 0 g/cm^3). All simulations were run in the NVT (constant number of particles N, volume V, and temperature T) ensemble at 253 K. For each system, more than 200 ps simulations were performed with a time step of 2 fs. The final 100 ps equilibrated trajectory for each system was used for further analysis. To better understand the H-bonding interaction between PVA chains, we performed H-bonds analysis using VMD.

Supplementary Note 3. The ionic-conductivity of suppressed cryogels

The conductivity of the suppressed cryogels was obtained by electrochemical impedance spectroscopy. Take a cryogel with uniform thickness and certain area (10 mm in length, 10 mm in width, and 2 mm in thickness), clamp it with two platinum sheets (10 mm in length, 10 mm in width, and 1 mm in thickness), and connect it to an electrochemical workstation (PGSTAT204, AUT52785). Among them, the reference electrode clip and the counter electrode clip of the electrochemical workstation are clamped on the same platinum sheet, and the working electrode clip of the electrochemical workstation is clamped on another platinum sheet. The impedance test frequency is 0.01~100 KHz, and the initial voltage is 10 mV. Conductivity is calculated by the following equation:

$$\sigma = \frac{Z'}{Z'^2 + Z''^2} \times \frac{d}{S} \qquad (1)$$

where Z' and Z'' are the real and imaginary parts of the impedance, S and d is the area

and thickness of the suppressed cryogels.

The resistance changes of suppressed cryogels during self-healing process are investigated. Under the voltage of 0.6 V and the frequency of 30 KHz, the suppressed cryogels are connected to the impedance analyzer (WK 6500B) through copper wires. The suppressed cryogels were cut into two pieces by a scalpel and then they were put together, where the resistance changes in the whole process were recorded.

Supplementary Note 4. Electrochemical measurements of the suppressed cryogel electrodes

The electrochemical properties of the suppressed cryogel electrodes were investigated by electrochemical workstations (PGSTAT204, AUT52785) using a three-electrode configuration. the suppressed cryogel electrodes (4 cm^2), platinum sheets (3 cm^2), and AgCl were used as working, counter, and reference electrodes, respectively. CV curves were recorded at a scan rate of 0.05, 0.1, 0.2, 0.5, 1 V s^{-1} with an electrochemical window of 0–0.7 V.

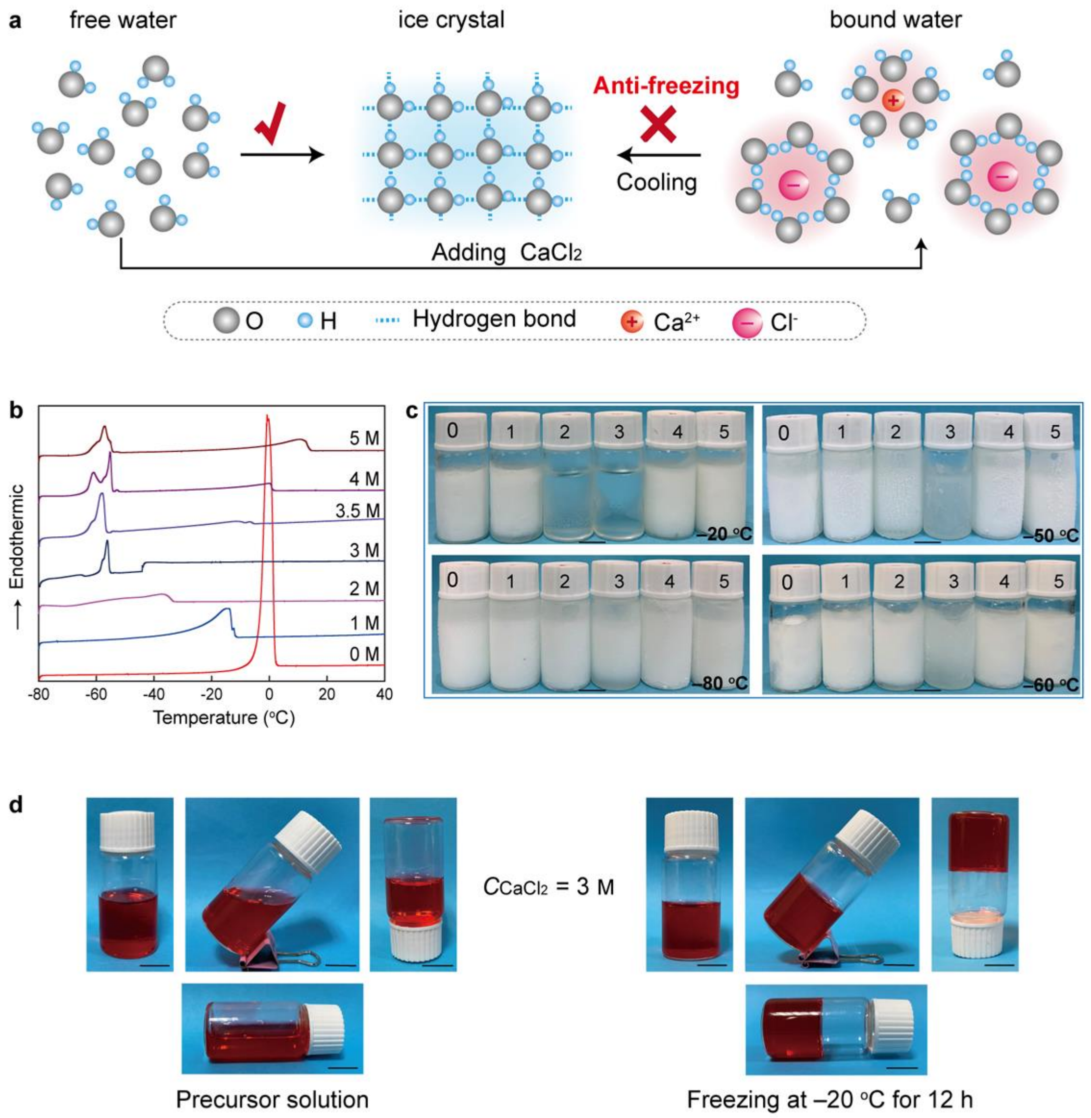

Supplementary Fig. 1 The freezing behaviors of precursor solution with anti-freezing salts. a The schematic illustration of molecular interaction between $CaCl_2$ and water molecules, indicating anti-freezing mechanism. **b** The heating runs of DSC curves for PVA/$CaCl_2$/H_2O solution (C_{PVA} = 14 wt%) with various $CaCl_2$ concentrations (M). **c** The freezing states of mixture solutions at –20, –50, –60, and –80 °C, respectively. **d** The flowing state of precursor solution and its non-flowing state after freezing at –20 °C for 12 h. Scale bars: 10 mm in (**c**–**d**).

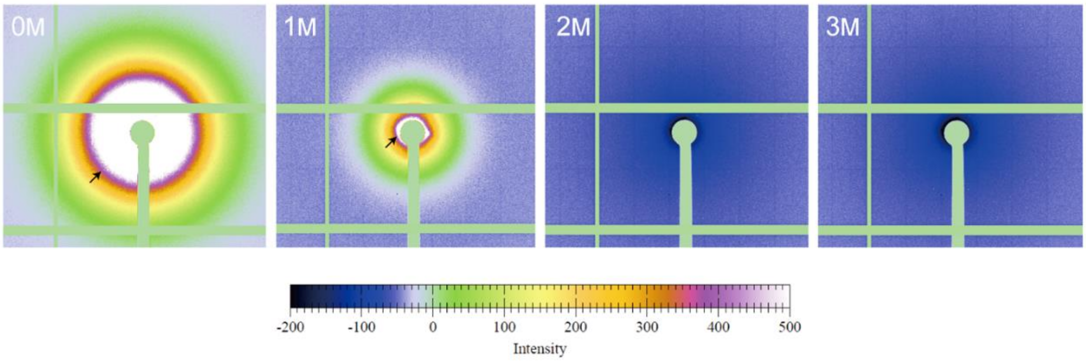

Supplementary Fig. 2 The SAXS patterns of cryogels (0, 1 M) and suppressed cryogels (2, 3 M).

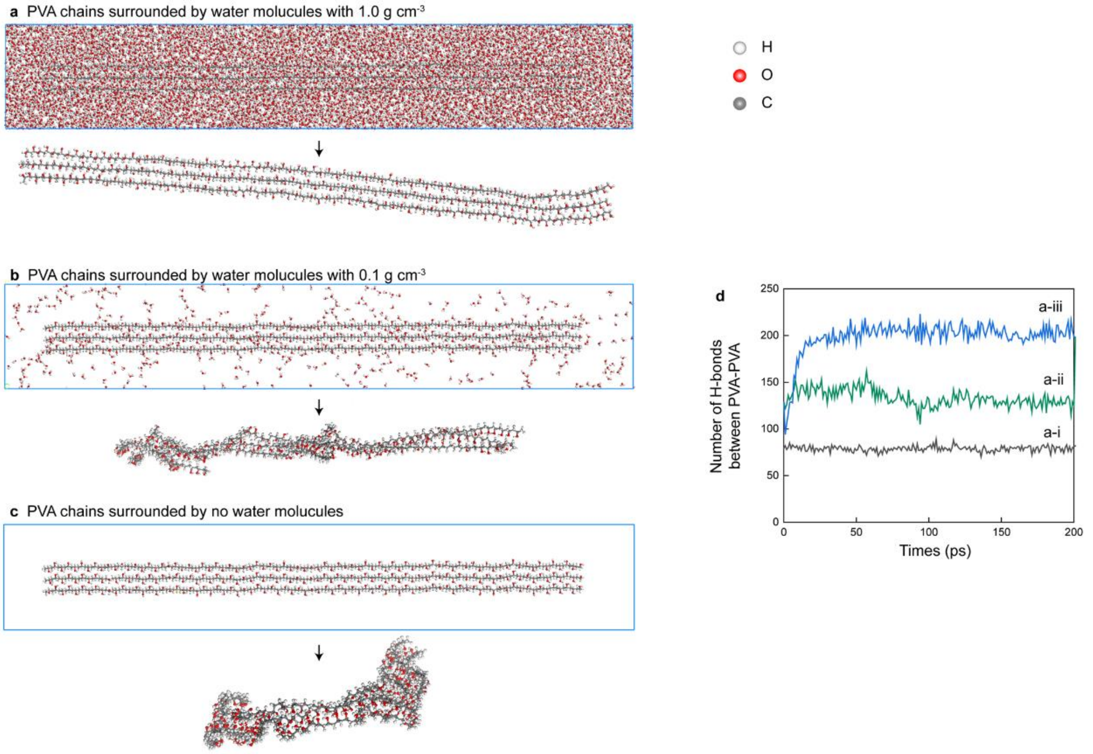

Supplementary Fig. 3 Molecular dynamics simulations of PVA chains surrounding by various number of water molecules. a–c The conformation of PVA chains with various number of water molecules. According to the simulation results of polymer conformation for cryogels, the PVA chains become entangled due to less water molecules, while the PVA chains of suppressed cryogels maintain a more extended conformation with enough water molecules. **d** The number of H-bonds between PVA-PVA chains. To better understand the H-bonding interaction between PVA chains, we performed H-bonds analysis using VMD. In the equilibrated trajectories, PVA chains with more interacting water molecules (suppressed cryogels) favor less H-bonds than those of conventional cyogels with small amount of interacting water molecules, which is consistent with FTIR results.

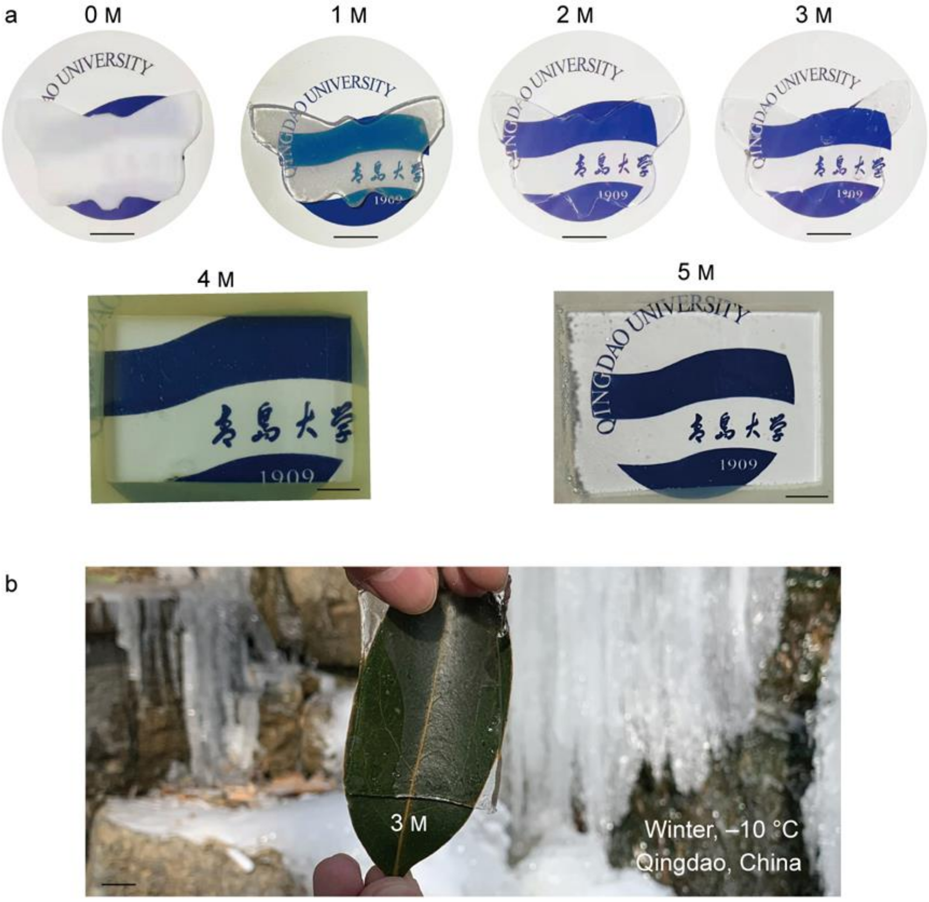

Supplementary Fig. 4 The transparency of cryogels with various concentrations of $CaCl_2$ in precursor solution. a The transparency of cryogels (0, 1 M), suppressed cryogels (1 , 2 and 3 M) and unstable cryogels (4, 5 M) with thickness of 2 mm. **b** The state of suppressed cryogels (3 M) put in cold winter in Qingdao, China. Scale bars: 10 mm in (**a, b**).

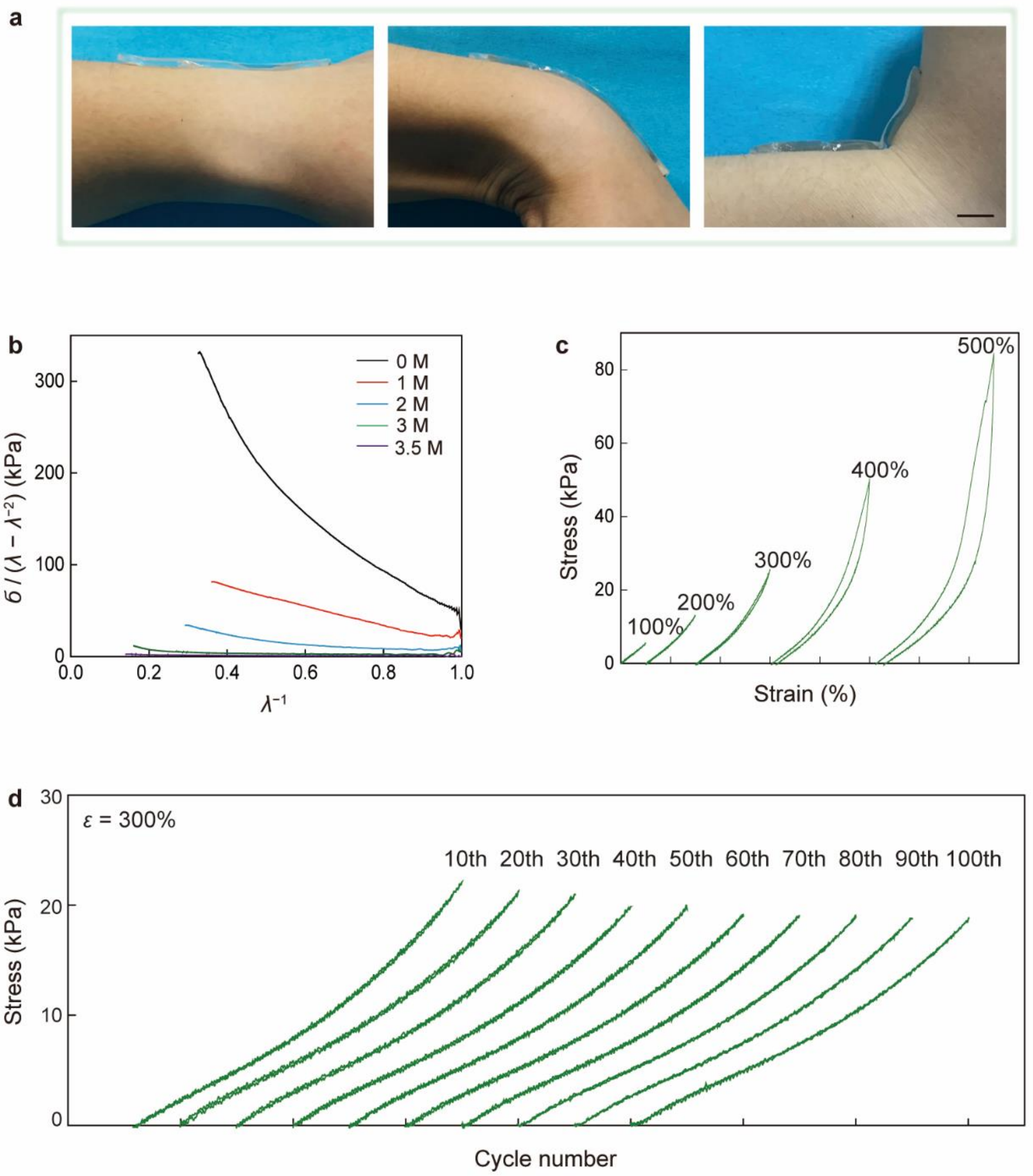

Supplementary Fig. 5 The mechanical behaviors of suppressed cryogels. a The suppressed cryogels (3 M) deform cooperatively with elbow (We have an informed consent from the research participant depicted in these figures). Scale bars: 20 mm. **b** Mooney-Rivlin profiles of tensile data (*X* and *Y* axes represent the elongation ratio $\lambda = \varepsilon_f + 1$ and reduced stress, respectively.) **c** Loading-unloading curves of suppressed cryogels with various strain. **d** Loading-unloading curves of suppressed cryogels at strain of 300% for 100 cycles.

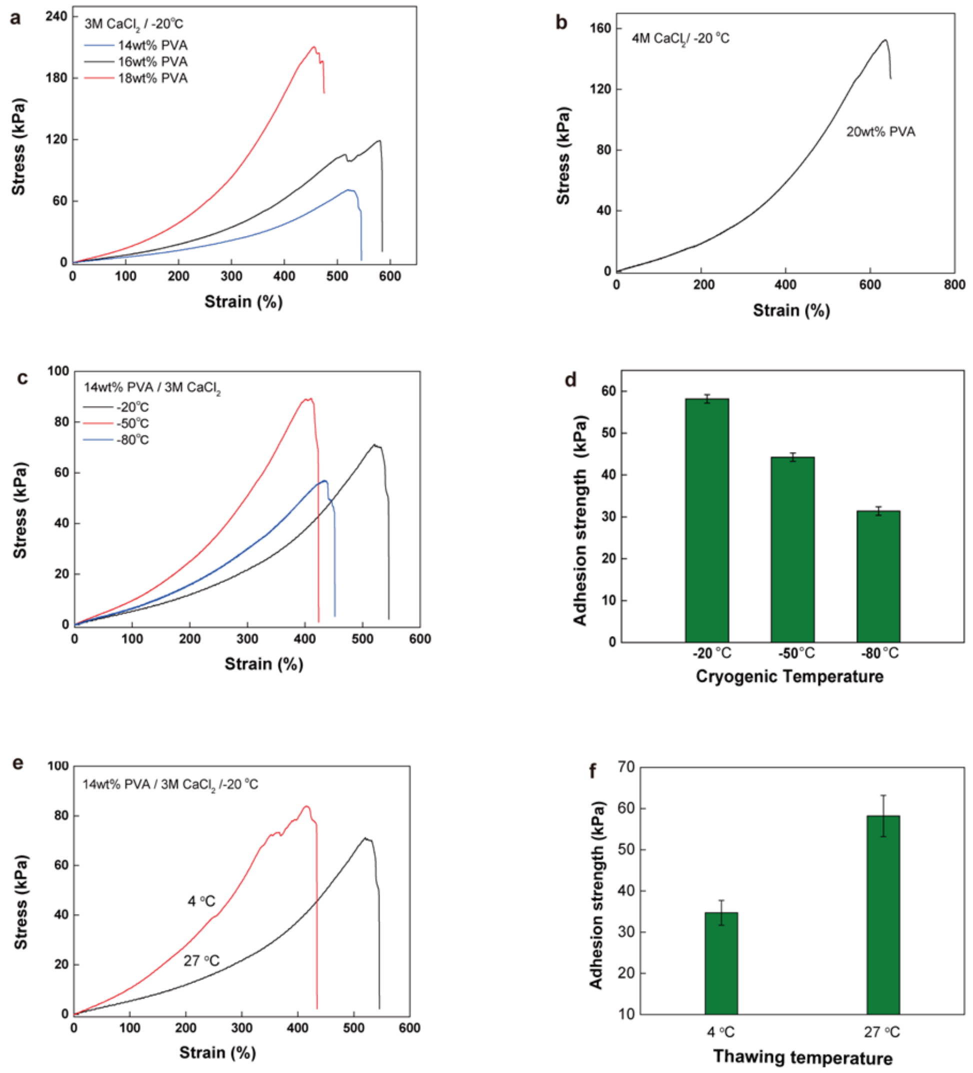

Supplementary Fig. 6 The variations of tensile and self-adhesive behaviors with fabricating parameters. a, b The variation of stress-strain curves for suppressed cryogels with PVA concentration. **c, d** The variation of stress-strain curves (**c**) and adhesion strength (**d**) with cryogenic temperature. **e, f** The variation of stress-strain curves (**e**) and adhesion strength (**f**) with thawing temperature. Error bars = standard deviation (n = 6) in (**d**, **f**).

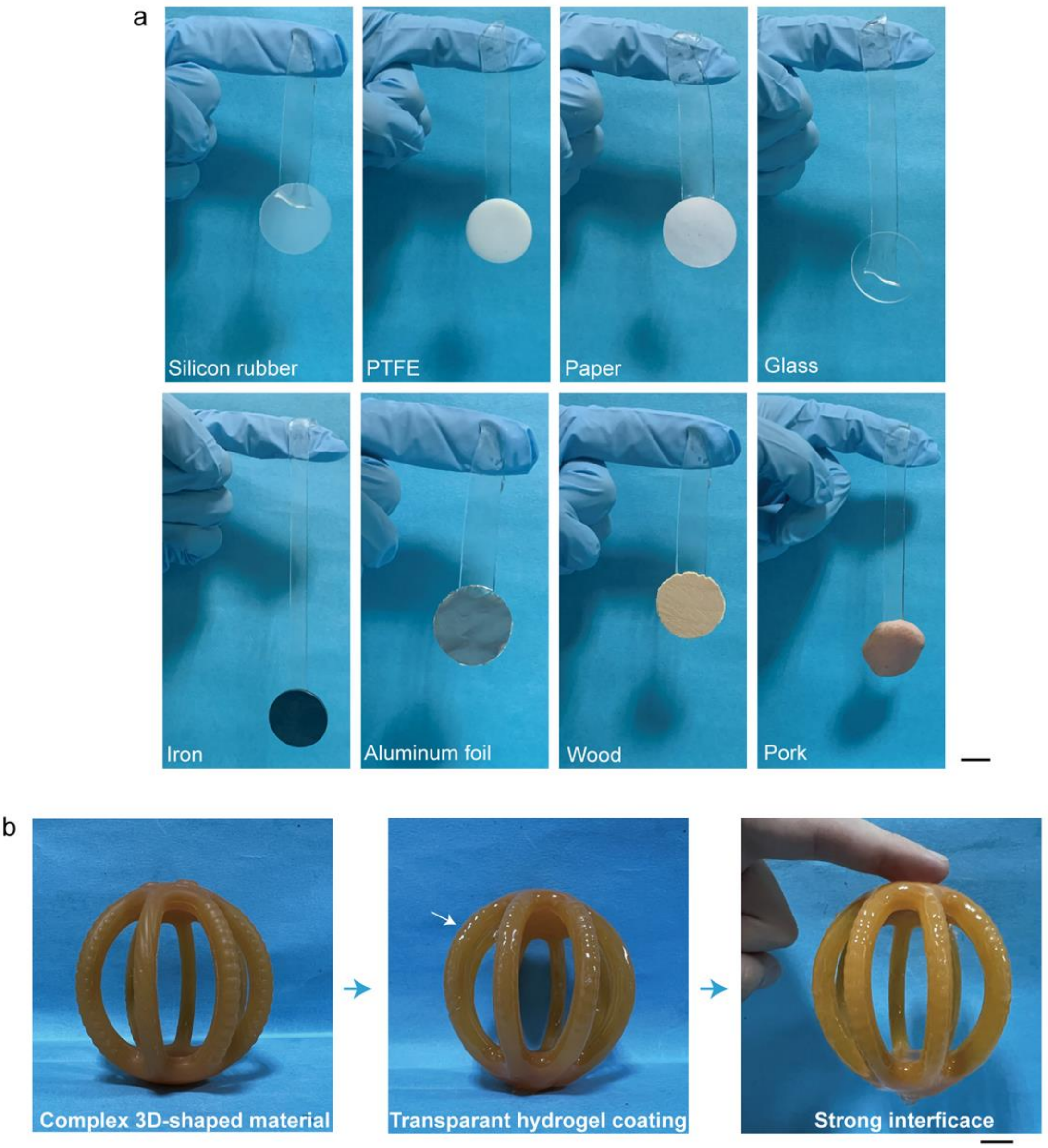

Supplementary Fig. 7 The self-adhesive nature of suppressed cryogels. a The suppressed cryogels adhered to various kinds of substrates. **b** Transparent, adhesive and ionic-conductive hydrogel coating on complex 3D-shaped materials. Scale bars: 10 mm in (**a, b**).

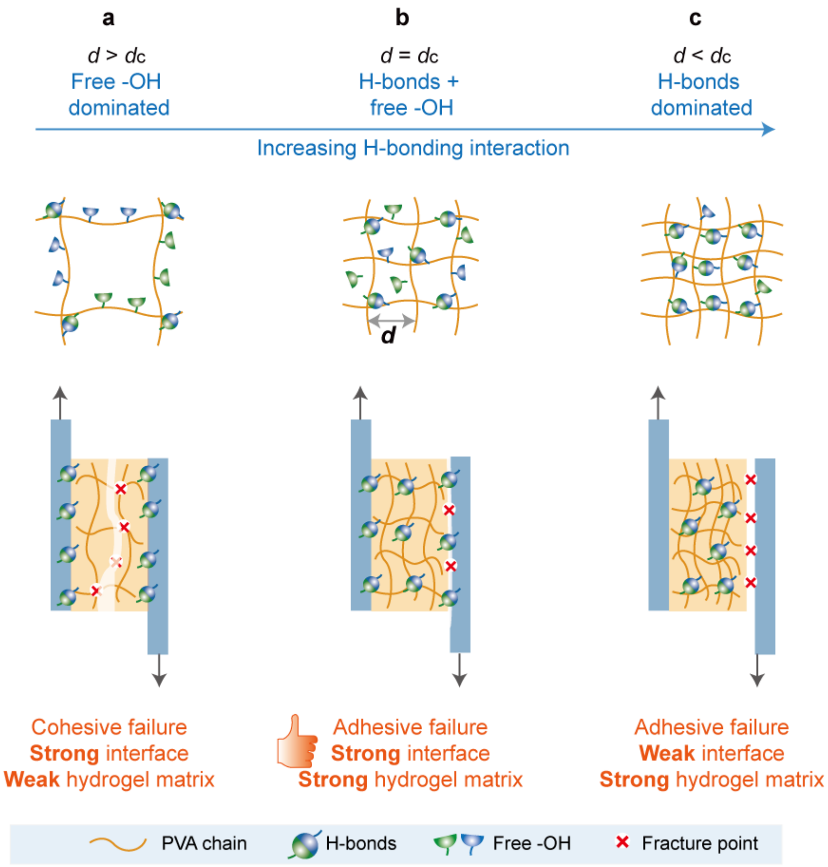

Supplementary Fig. 8 The schematic illustration of self-adhesiveness and its variation with the distance between adjacent chains *d*. a $d > d_c$. **b** $d = d_c$. **c** $d < d_c$.

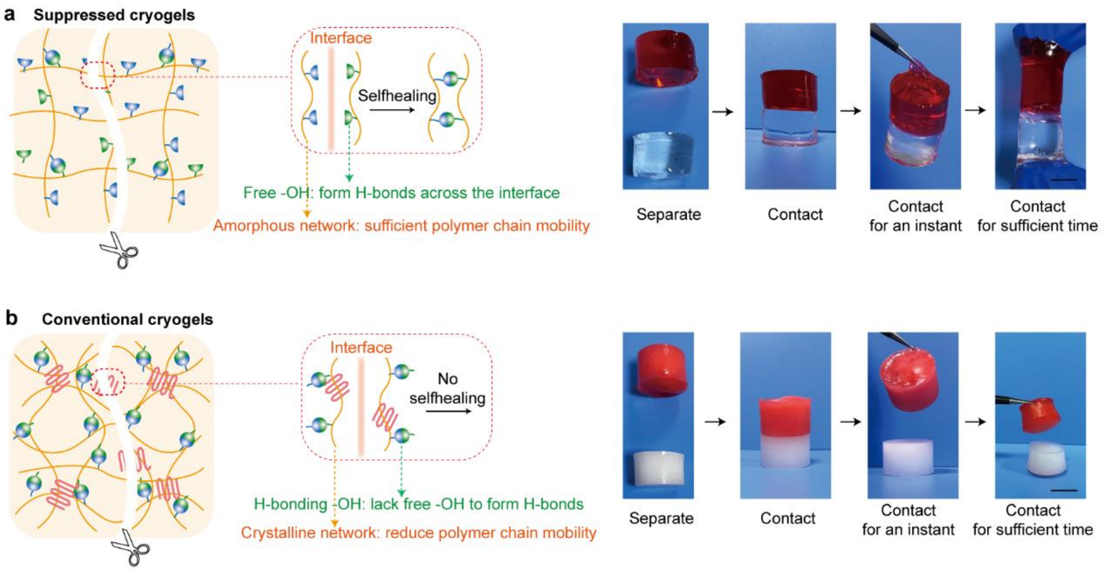

Supplementary Fig. 9 The schematic mechanism and the corresponding appearance of self-healing for suppressed cryogels (**a**) and conventional freeze-thawed cryogels (**b**). Scale bars: 20 mm.

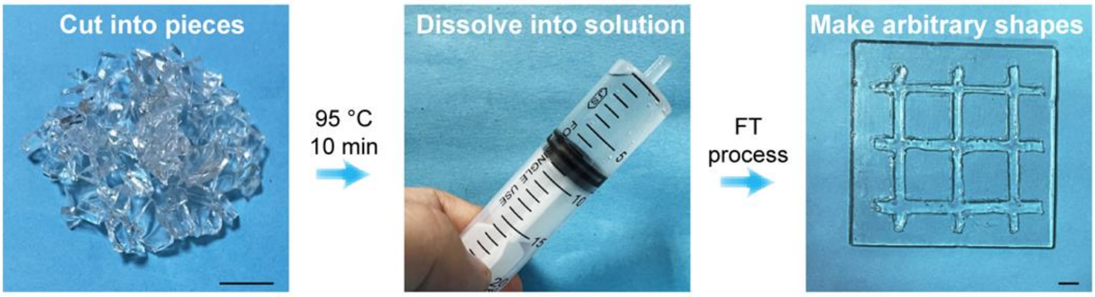

Supplementary Fig. 10 Photographs showing reprocessing ability into arbitrary shapes for suppressed cryogels (C_{CaCl_2} = 3 M). Scale bars: 10 mm.

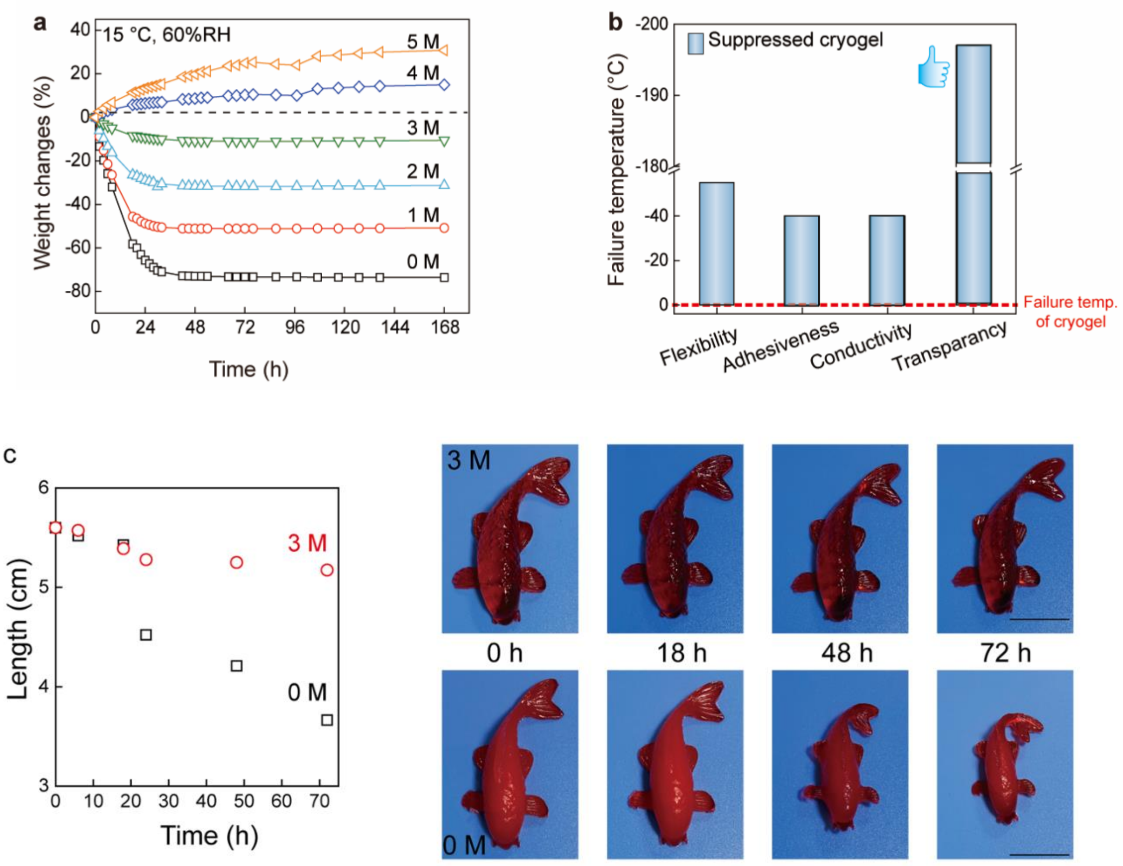

Supplementary Fig. 11 The stability of suppressed cryogels in comparison with conventional freeze-thawed cryogels. a Weight changes with time for cryogels and suppressed cryogels with various $CaCl_2$ concentrations under temperature of 15 °C and relative humidity of 60% *RH*. **b** Failure temperature of multiple properties for cryogels and suppressed cryogels (C_{CaCl_2} = 3 M). **c** The size changes and the corresponding appearance of both hydrogels with five days. Scale bars: 20 mm.

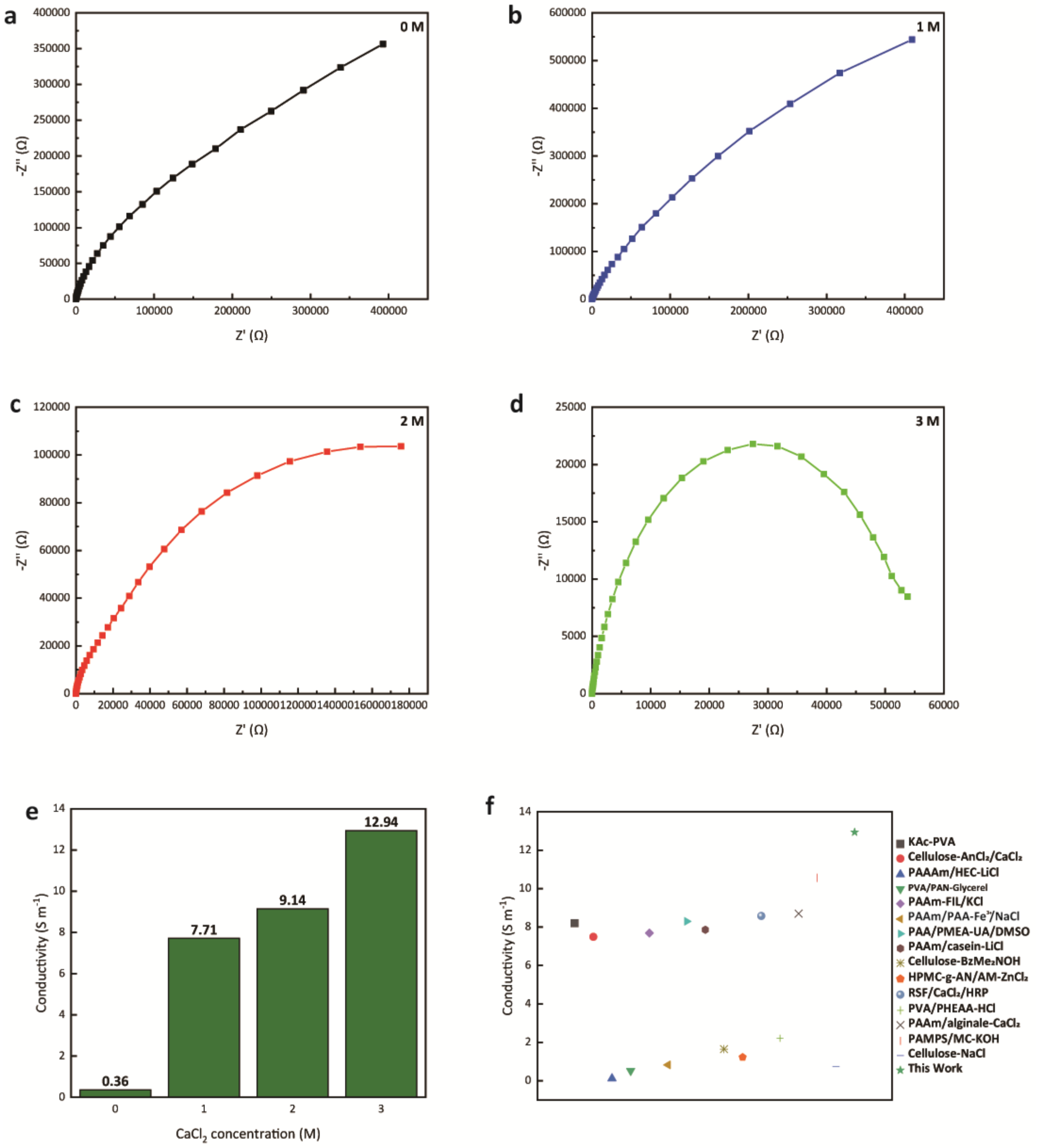

Supplementary Fig. 12 The conductivity of suppressed cryogels. a–d Electrochemical Impedance Spectroscopy of the suppressed cryogels. Using the electrochemical workstation, electrochemical impedance spectroscopy tests were performed on the cryogels containing different $CaCl_2$ concentrations. The frequency of impedance test is 0.01–100 KHz, and the initial voltage is 10 mV. **e** The variation of conductivity for the cryogels with $CaCl_2$ concentrations. **f** Comparison of conductivity between suppressed cryogels and reported ionic hydrogels.

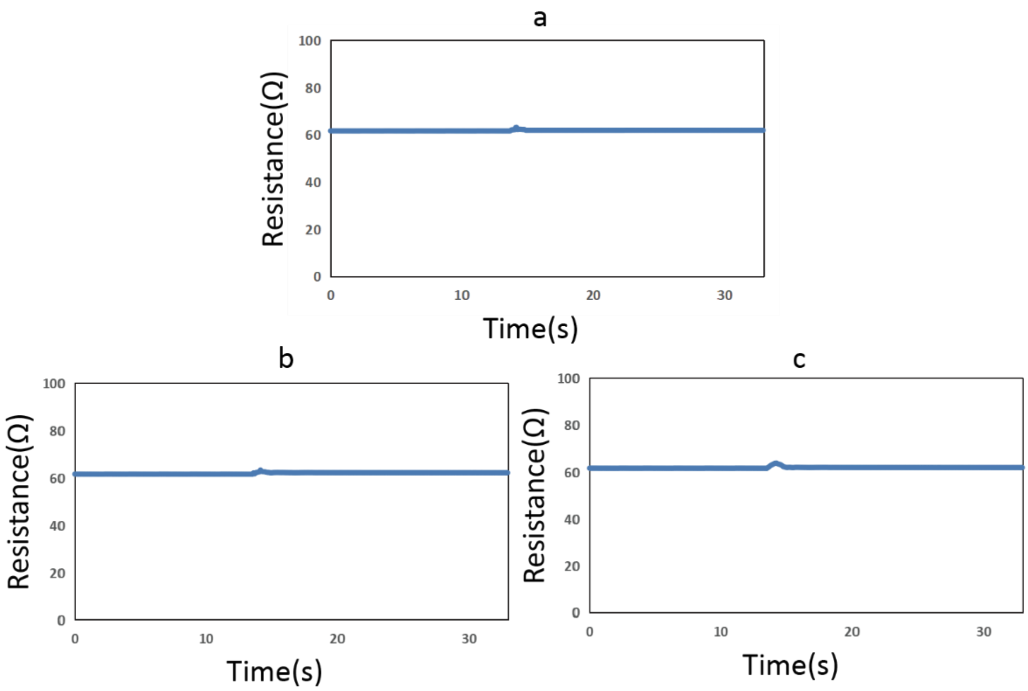

Supplementary Fig. 13 The resistance changes of hydrogels with the same size under different cutting conditions. From **a** to **c**, the hydrogels were cut with decreasing speed (5, 1, 0.1 m/s) using the same utility knife. The data show that with the decreasing speed, the resistance of the hydrogel changes more obviously during cutting.

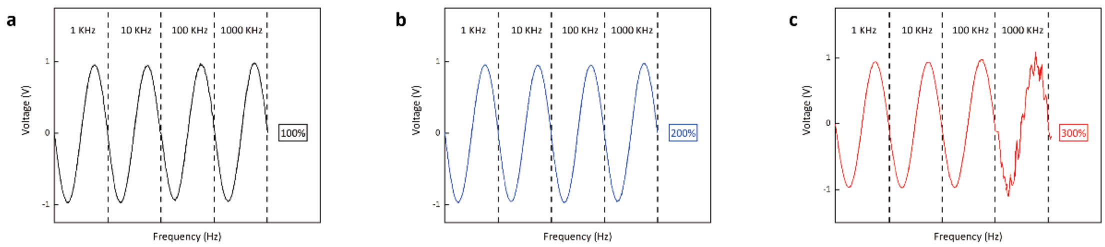

Supplementary Fig. 14 Information transmission performance of artificial nerve fibers when stretched at low temperature. At –45 °C, when the tensile deformation of the artificial nerve fibers is 100% (**a**), 200% (**b**) and 300% (**c**), the AC signals of the output signal V_1 at different amplitudes and frequencies are monitored.

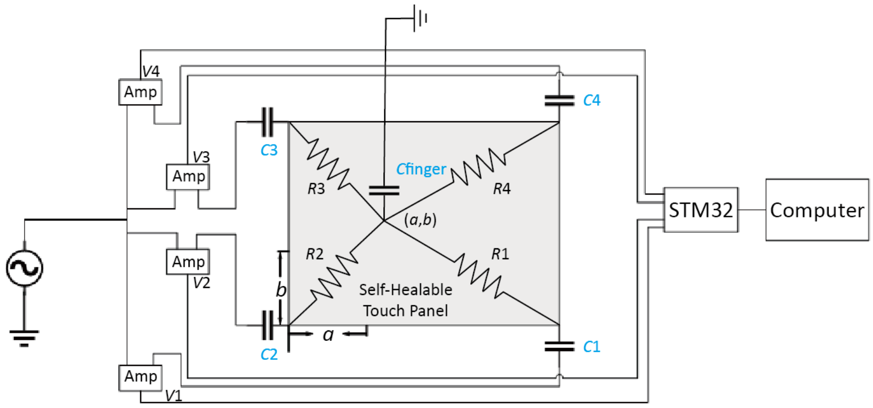

Supplementary Fig. 15 Simulation of the entire module when the hand is in contact with the hydrogel. When the finger touches the panel, a closed circuit is formed due to that the finger is grounded. In this way, the current flows from four corners of the panel to the contacts, and the panel is virtually divided into four resistance parts via the contacts. In the circuit, four virtual resistors are connected in parallel, which is connected to the capacitor in series through the fingers. The normalized distance of *a* and *b* are introduced when the position of the suppressed cryogel is converted to the computer. *V*1, *V*2, *V*3 and *V*4 are the voltage after the current is amplified by four operational amplifiers. *C*1, *C*2, *C*3 and *C*4 are the electric double layer capacitance between platinum sheet and the suppressed cryogel. *C*finger is the capacitance generated by the human hand in contact with cryogel. *R*1, *R*2, *R*3, and *R*4 are the resistance between the touch point and the four corners of the cryogel when the human hand touches the cryogel.

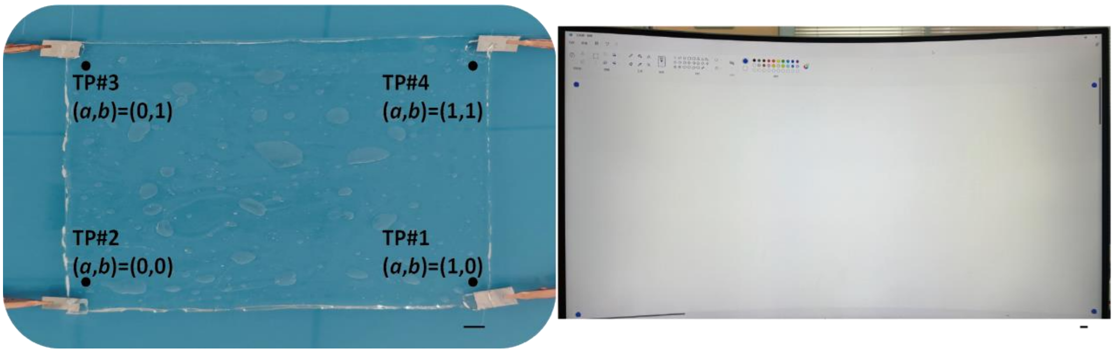

Supplementary Fig. 16 The comparison of specific position between the self-healing touch pad and the computer. When the four points TP#1, TP#2, TP#3, and TP#4 on the touch panel is touched in turn, they can be displayed on the computer accordingly. The normalized distance of *a* and *b* are introduced when the position of the suppressed cryogel is converted to the computer. Scale bars: 30 mm.

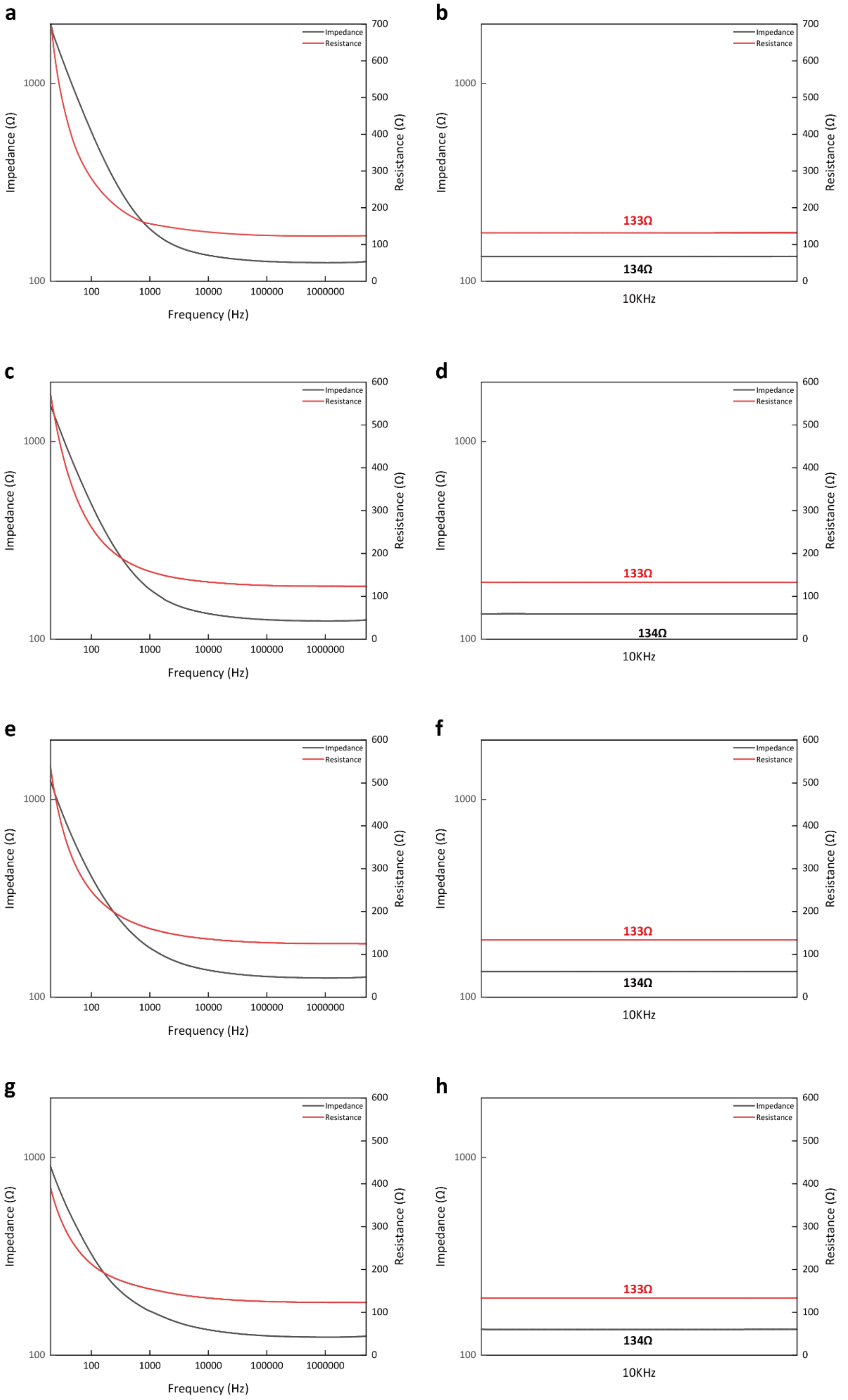

Supplementary Fig. 17 The impedance and resistance changes of the pressure

sensor under sweep and fixed frequency. The impedance and resistance changes of the pressure sensor when the AC amplitude is 0.5 V (**a**), 0.6 V (**c**), 0.8 V (**e**), 1 V (**g**) with the frequency range of 20 Hz–5 MHz. The impedance and resistance changes of the pressure sensor within 36 s when the AC amplitude is 0.5 V (**b**), 0.6 V (**d**), 0.8 V (**f**), 1 V (**h**) with the frequency of 10 KHz.

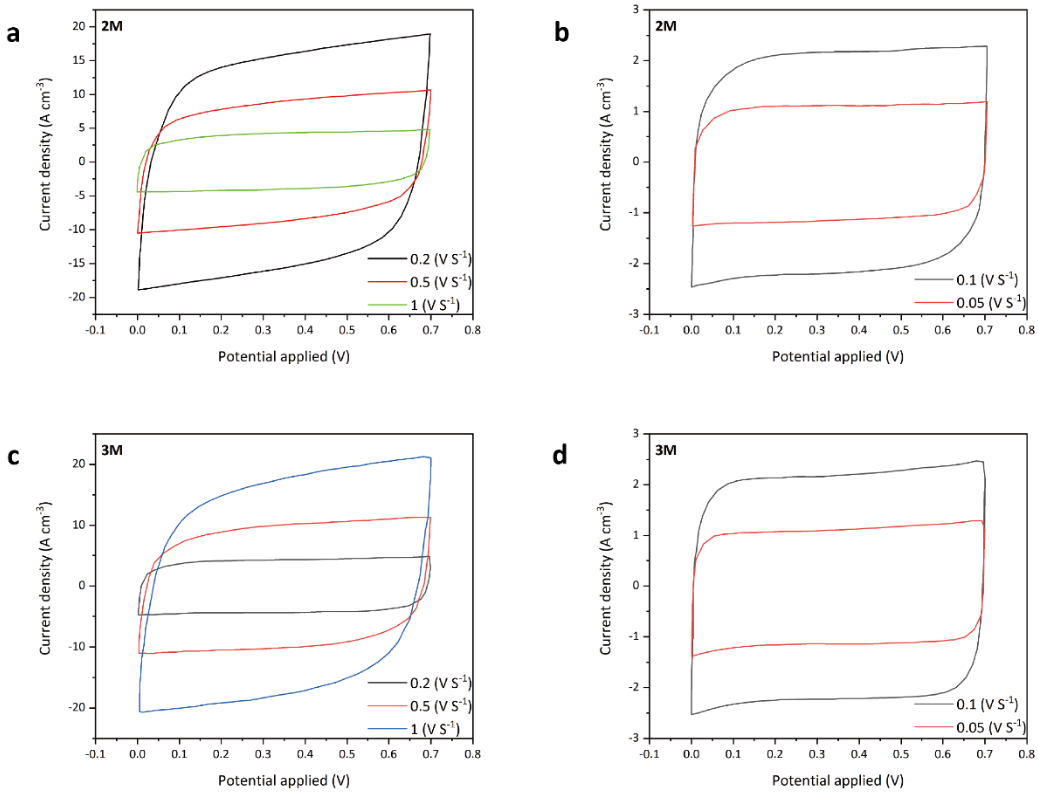

Supplementary Fig. 18 Electrochemical performance of the suppressed cryogel electrodes with different $CaCl_2$ concentrations. The test was performed at 20 °C with a three-electrode configuration. **(a–d)** Cyclic voltammograms of the suppressed cryogel electrode with a scan voltage of 0–0.7 V and scan rates of 0.05, 0.1, 0.2, 0.5, and 1 V S^{-1}.

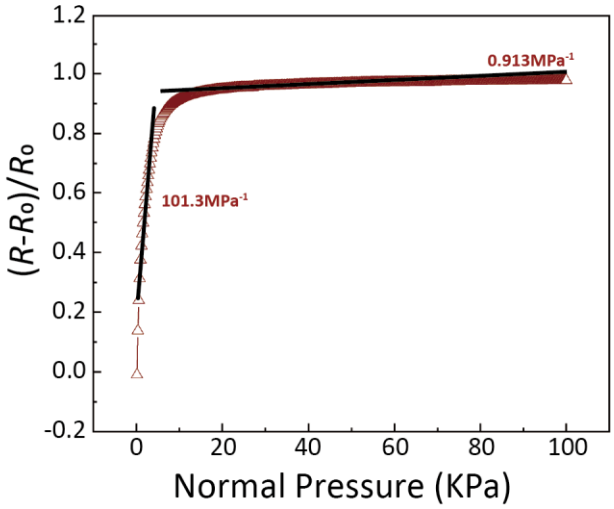

Supplementary Fig. 19 Sensitivity of the pressure sensor.

The calculation is as follows:

$$S = \frac{\delta(\frac{R-R_0}{R_0})}{\delta P} \qquad (2)$$

Among them, S is the sensitivity, R_0 is the initial resistance, R is the resistance after the load is applied, and P is the value of the applied load.

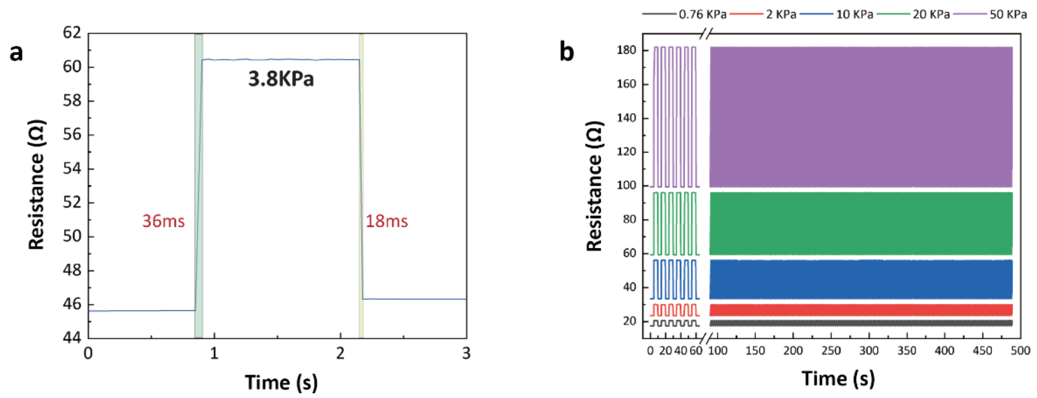

Supplementary Fig. 20 Response time, stability and repeatability of pressure sensors. a The response time of the pressure sensor (piezoresistive meter). The response time is 36 ms when loading 3.8 kPa. The response time is 18 ms when releasing the load. **b** Stability and repeatability of the pressure sensor (200 cycles) under pressure of 0.76 kPa (black), 2 kPa (red), 10 kPa (blue), 20 kPa (green) and 50 kPa (purple).

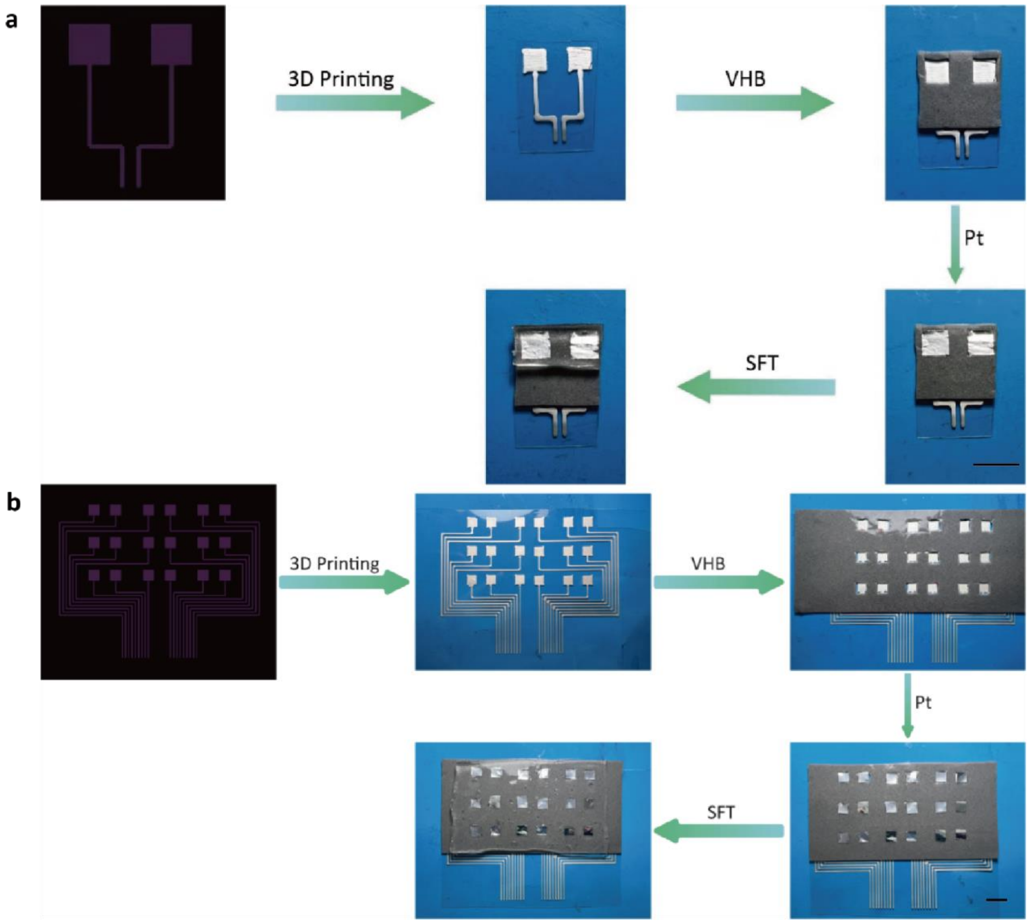

Supplementary Fig. 21 Fabrication process of pressure sensor and pressure sensor array. a Firstly, a 3D printer (Scientific 3) was used to print the designed electrode pattern on a flexible PET substrate. Secondly, a 3M tape of a specific shape was stuck on the PET substrate, then a platinum sheet was placed on the 3M tape, and finally the suppressed cryogel placed on the top layer. **b** Firstly, a 3D printer (Scientific 3) was used to print out the designed electrode array pattern on a flexible PET substrate. Secondly, a 3M tape of a specific shape was stuck on the PET substrate, then a platinum sheet was placed on the 3M tape, and finally the suppressed cryogel is placed on the top layer. Scale bars: 10 mm.

Supplementary references

1. Mahabole, M. P., Bahir, M. M., Kalyankar, N. V. & Khairnar, R. S. Effect of incubation in simulated body fluid on dielectric and photoluminescence properties of nano-hydroxyapatite ceramic doped with strontium ions. *J. Biomed. Sci. Eng.* **5**, 396–405 (2012).
2. Wu, S. et al. Tough, anti-freezing and conductive ionic hydrogels. *NPG Asia Mater.* **14**, 65 (2022).
3. Xu, Y., Rong, Q., Zhao, T. & Liu, M. Anti-freezing multiphase gel materials: bioinspired design strategies and applications. *Giant* **2**, 100014 (2020).
4. Morelle, X. P. et al. Highly stretchable and tough hydrogels below water freezing temperature. *Adv. Mater.* **30**, 1801541 (2018).
5. Zhang, X. et al. Inorganic salts induce thermally reversible and anti-freezing cellulose hydrogels. *Angew. Chem. Int. Ed.* **58**, 7366–7370 (2019).
6. Ren, Y. et al. Ionic liquid–based click-ionogels. *Sci. Adv.* **5**, eaax0648 (2019).
7. Liu, Z. et al. Poly(ionic liquid) hydrogel-based anti-freezing ionic skin for a soft robotic gripper. *Mater. Horiz.* **7**, 919–927 (2020).
8. Rong, Q., Lei, W., Huang, J. & Liu, M. Low temperature tolerant organohydrogel electrolytes for flexible solid-state supercapacitors. *Adv. Energy Mater.* **8**, 1–7 (2018).
9. Yang, Y. et al. Anti-freezing, resilient and tough hydrogels for sensitive and large-range strain and pressure sensors. *Chem. Eng. J.* **403**, 126431 (2021).
10. Duan, S. et al. Tendon-inspired anti-freezing tough gels. *iScience* **24**, 102989 (2021).
11. Wu, S. et al. Rapid and scalable fabrication of ultra-stretchable, anti-freezing conductive gels by cononsolvency effect. *EcoMat* **3**, e12085 (2021).
12. Yao, B. et al. Hydrogel ionotronics with ultra-low impedance and high signal fidelity across broad frequency and temperature ranges. *Adv. Funct. Mater.* **32**, 2109506 (2022).
