# Supplementary material for: Skin-like cryogel electronics from suppressed-freezing tuned polymer amorphization
Source: Nat Commun. 2023 Aug 17;14:5010. doi: 10.1038/s41467-023-40792-y (PMC10435499; doi:10.1038/s41467-023-40792-y)
Supplement: Supplementary file 3 — Description of Additional Supplementary Files [file 41467_2023_40792_MOESM3_ESM.pdf]

Description of Additional Supplementary Files

File Name: **Supplementary Movie 1**
Caption: Anti-freezing ability in liquid nitrogen

File Name: **Supplementary Movie 2**
Caption: Uniaxial stretching of suppressed cryogels and common cryogels

File Name: **Supplementary Movie 3**
Caption: Suppressed cryogels with ultrasoftness, transparency and self-adhesiveness

File Name: **Supplementary Movie 4**
Caption: Instantaneous self-healing of suppressed cryogels

File Name: **Supplementary Movie 5**
Caption: Stretchability of artificial nerve fibers

File Name: **Supplementary Movie 6**
Caption: The working ability of artificial nerve fibers in ultra-low temperature environment
